# A Metabolic Gene Signature to Predict Overall Survival in Head and Neck Squamous Cell Carcinoma

**DOI:** 10.1155/2020/6716908

**Published:** 2020-12-30

**Authors:** Zeng-Hong Wu, Yun Tang, Yue Zhou

**Affiliations:** ^1^Department of Otorhinolaryngology, Union Hospital, Tongji Medical College, Huazhong University of Science and Technology, Wuhan, Hubei, China; ^2^Department of Infectious Diseases, Union Hospital, Tongji Medical College, Huazhong University of Science and Technology, Wuhan 430022, China; ^3^Department of Critical Care Medicine, Union Hospital, Tongji Medical College, Huazhong University of Science and Technology, Wuhan 430022, China

## Abstract

**Background:**

Head and neck squamous cell carcinoma (HNSCC) is a common malignancy that emanates from the lips, mouth, paranasal sinuses, oropharynx, larynx, nasopharynx, and from other pharyngeal cancers. The availability of high-throughput expression data has made it possible to use global gene expression data to analyze the relationship between metabolic-related gene expression and clinical outcomes in HNSCC patients.

**Method:**

In this study, we used RNA sequencing (RNA-seq) data from the cancer genome atlas (TCGA), with validation in the GEO dataset to profile the metabolic microenvironment and define potential biomarkers for metabolic therapy.

**Results:**

We extracted data for 529 patients and 327 metabolic genes (198 upregulated and 129 downregulated genes) in the TCGA database. Carbonic anhydrase 9 (CA9) and CA6 had the largest logFCs in the upregulated and downregulated genes, respectively. Our Cox regression model data showed 51 prognostic-related genes with lysocardiolipin acyltransferase 1 (*LCLAT1*) and choline dehydrogenase (*CHDH*) being associated with the highest risk (HR = 1.144, 95% CI = 1.044 ~ 1.251) and the lowest risk (HR = 0.580, 95% CI = 0.400 ~ 0.839) in HNSCC, respectively. We next used the ROC curve to evaluate whether the differentially expressed metabolic-related genes could serve as early predictors of HNSCC. The findings showed an AUC of 0.745 and 0.618 in the TCGA and GEO analysis, respectively. Besides, the ability for the genes to predict clinicopathological HNSCC status was analyzed and the data showed that the AUC for age, gender, grade, stage, T, M, and N was 0.520, 0.495, 0.568, 0.606, 0.577, 0.476, and 0.673, respectively, in the TCGA dataset. On the other hand, the AUC for age, gender, stage, T, M, N, smoking, and HPV16-pos was 0.599, 0.531, 0.622, 0.606, 0.616, 0.550, 0.614, 0.519, and 0.397, respectively, in the GEO dataset.

**Conclusion:**

Taken together, our study unearths a novel metabolic gene signature for the prediction of HNSCC prognosis based on the TCGA dataset. Our signature might point out the metabolic microenvironment disorders and provides potential treatment targets and prognostic biomarkers.

## 1. Background

Head and neck squamous cell carcinoma (HNSCC) is a malignancy that originates from the lips, mouth, paranasal sinuses, larynx, nasopharynx, and from other pharyngeal cancers [1]. The HNSCC is the sixth most common type of malignant tumors, with more than 655,000 new cases and 90,000 deaths every year [2]. Smoking, drinking, and human papillomavirus (HPV) infections are considered risk factors for the occurrence and development of HNSCC [3]. Worryingly, due to the lack of early manifestation of symptoms or diagnosis, local recurrence, and metastasis, the 5-year survival rate still lags at below 50% [4]. The occurrence and development of HNSCC are complex processes that are mediated by multiple molecules and pathways. Kim et al. reported the mechanistic and functional roles of *CXCR7* as a key regulator of oncogenic TGF-*β*1/Smad2/3 signaling in HNSCC [5]. In addition, Hsu et al. defined the oncogenic driver role of atypical cadherin 1 (*FAT1*) in the mediation of proliferation, cell-death evasion, and chemoresistance in oral squamous cell carcinoma (OSCC) [6]. Moreover, another study revealed that the expression of E6 and E7, the HPV virus oncogenes, inactivates p53 and retinoblastoma (RB), respectively [7]. However, there is a wide spectrum of histological tumor markers for HNSCC and multiple anatomical sites. Therefore, it is possible to identify more valuable HNSCC drug targets by screening for changes in gene function networks linked to tumor formation and progression.

The activation of oncogenes and lack of tumor suppressors contribute to metabolic reprogramming in cancer, leading to improved nutrient uptake that feed biosynthetic pathways [8]. In the 1920s, Otto Warburg first reported that tumors took up distinctly more levels of glucose compared with normal tissues, indicating that these cells were biased towards shuttling glucose via the glycolytic pathway [9]. Recent studies have shown that immune cells have unique metabolic characteristics that affect their immune function. For example, macrophage polarization is associated with unique metabolic characteristics related to iron, energy, and lipid metabolism [10, 11]. Whereas a number of studies have investigated that the prognostic role of these metabolic genes in cancer, data on the role, and mechanism of metabolism still remains scant. In their studies, Hu et al. found that mutationally activated *KRAS* robustly increased the glutathione biosynthesis and intracellular cystine level in lung adenocarcinoma [12]. On the other hand, Yoo et al. found that the *SLC1A5* variant is a mitochondrial glutamine transporter used for metabolic reprogramming of pancreatic cancer, and the knockout or overexpression of the *SLC1A5* variant alters the growth of cancer cells and tumors, thus, supporting carcinogenesis [13]. There is, however, no data on systematic assessment of the metabolic-related genes that could reliably predict the overall survival (OS) in HNSCC patients or characterize the patient response to immunotherapy. The availability of high-throughput expression data has made it feasible to utilize global gene expression data to analyze the relationships between the metabolic-related gene expression and clinical outcomes in HNSCC patients. In this study, we used RNA sequencing (RNA-seq) data from The Cancer Genome Atlas (TCGA) with validation from the Gene Expression Omnibus (GEO) dataset to profile the metabolic molecular microenvironment and assess their importance as biomarkers for metabolic therapy.

## 2. Methods

### 2.1. Data Collection

We extracted RNA-seq data for HNSCC patients from TCGA (http://www.cancergenome.nih.gov), a web-based resource that provides a user-friendly interface and depository for mRNA expression data. We validated all the data from the GSE65858 data set obtained from the GEO database and then extracted all the metabolic-related genes contained in the Gene set enrichment analysis (GSEA) database. One millionth transcript normalization and log2 transformation were used for expression profiling. The selection of metabolic-related genes for prognostic analysis was not only consistent with the expression patterns in the TCGA cohort but also listed in the GSE65858 data set.

### 2.2. Development of the Metabolic-Related Prognostic Gene Signature

Lasso-penalized Cox regression and Univariate Cox regression analyses were used to build the metabolic-related prognostic gene signature [14]. The signature was defined as risk score = (coefficient_mRNA1_ × expression of mRNA1) + (coefficient_mRNA2_ × expression of mRNA2) + ⋯+(coefficient_mRNAn_ × expression mRNAn). The related clinical data for HNSCC patients were also downloaded and evaluated. Based on the median, we denoted the data as either low-risk (<median number) or high-risk (≥median number) group. We used Kaplan–Meier survival analysis to analyze the survival rate for both the study and control groups.

### 2.3. Building and Validating a Predictive Nomogram

Here, we developed a nomogram [15] for the prediction of the occurrence of cancer events, such as recurrence or death. We then used the time-dependent receiver operating characteristic (ROC) curve to assess the predictive accuracy of the developed prognostic signatures for patients with HNSCC. Univariate and multivariate Cox regression analyses were employed to analyze the relationship between immune-related genes and clinicopathological manifestations.

### 2.4. External Validation of the Prognostic Gene Signatures

We downloaded the validated GSE65858 dataset in the GEO database. Following the assessment of the risk scores for the patients with genetic characteristics and carrying out the ROC analysis as well as the Kaplan-Meier analysis, we robustly demonstrated the similarity between the constructed nomogram and the TCGA-HNSCC cohort. To understand the mechanisms underlying defining the gene signatures in the Kyoto Encyclopedia of Genes and Genomes (KEGG), we used GSEA to search for rich terms in C2 in the TCGA-HNSCC or GSE65858 cohort. A *P* < 0.05 and a false discovery rate *q* < 0.25 were considered to be statistically significant. The mRNA expression level (Oncomine and TIMER database) and protein expression profile (The Human Protein Atlas database) further verified the expression of the genes included in the prognostic signatures. We then used CBioportal to study genetic alterations in the gene signatures.

### 2.5. Statistical Analysis

We filtered the data to ensure the complete exclusion of any sample with missing values. We used the Benjamini–Hochberg's method to convert the *P* values to FDR. Data were analyzed using R (version 3.5.3) and R Bioconductor software packages. We used Perl language for data matrix and data processing. A *P* value of 0.05 was considered significant.

## 3. Results

### 3.1. Development and Verification of the Prognostic Metabolic Gene Signatures

A total of 529 patients in the TCGA database and 327 metabolic genes (198 upregulated genes with the largest logFC of *CA9* and 129 downregulated genes with the largest logFC of *CA6*; Table S1) were used to model the prognostic signature for the HNSCC. The validating GSE65858 dataset contained 270 HNSCC tissue samples. Our Univariate Cox regression analysis showed 51 survival-related genes, with *LCLAT1* being associated with the highest risk (HR = 1.144, 95% CI = 1.044 ~ 1.251) and *CHDH* denoting the lowest risk (HR = 0.580, 95% CI = 0.400 ~ 0.839) (Table 1). The Lasso-penalized Cox analysis filtered 30 genes (*HEXB*, *ACAT1*, *GNPDA1*, *POLE2*, *SMS*, *AGPS*, *PYGL*, *ACAA1*, *MTHFD2*, *PLCB3*, *POLD2*, *KYNU*, *ENTPD1*, *LCLAT1*, *DNMT1*, *ADK*, *ADA*, *PAFAH1B2*, *PLA2G2D*, *DGKQ*, *ADH7*, *ACOX3*, *ASNS*, *HPRT1*, *ATIC*, *PRPS1*, *NADSYN1*, *RDH11*, *HADHB*, and *PIP4K2A*) used to build the prognostic model, and then we calculated the risk scores (Table 2). The samples were then divided into high- and low-risk groups using the median risk score value as a cut-off.

### 3.2. Survival Results and Multivariate Examination

**Figure 1:**
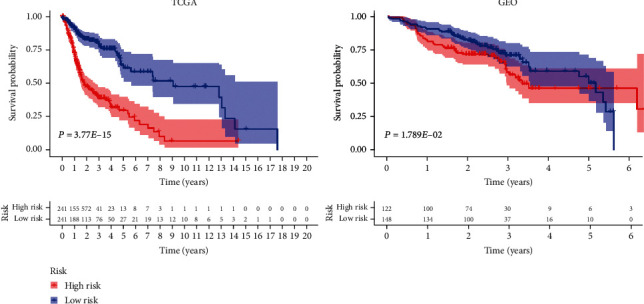
Overall survival (OS) analysis demonstrated that HNSCC with high-risk group had a more terrible prognosis than that with low-risk group (*P* < 0.01).

**Figure 2:**
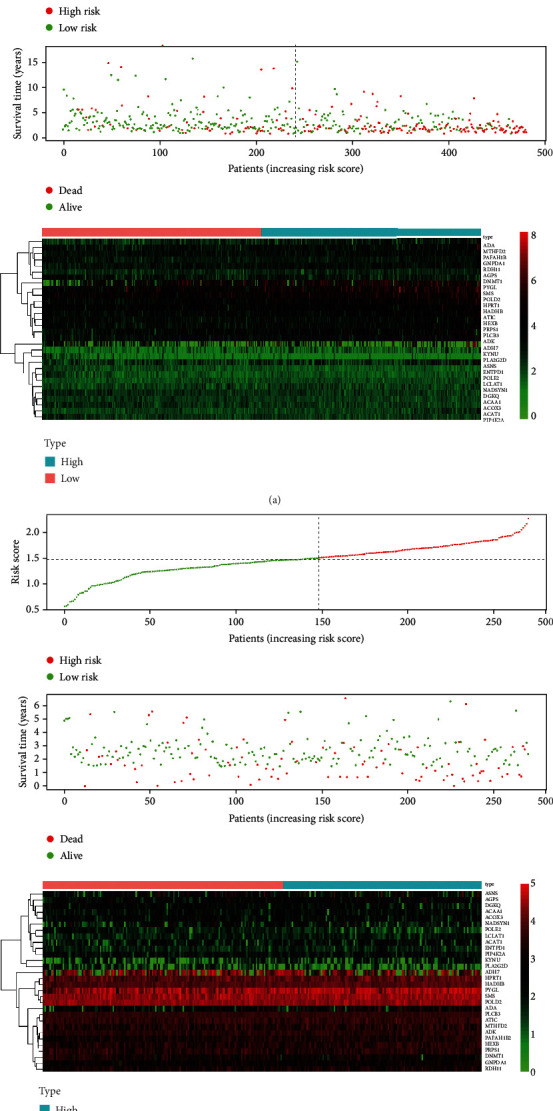
Detailed prognostic signature information of HNSCC groups is visualized.

**Figure 3:**
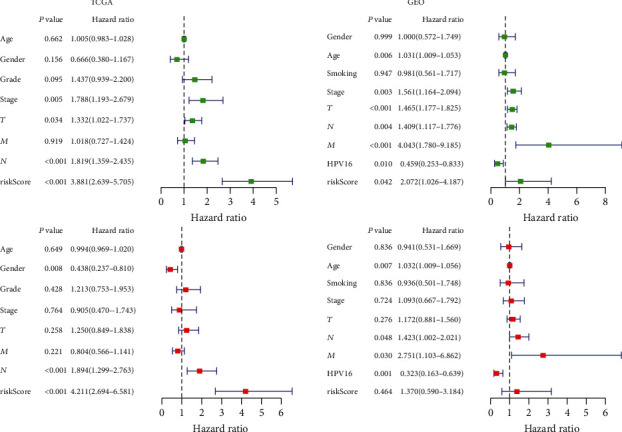
The result of univariate and multivariate Cox regression analysis showed that our prognostic model is an independent prognostic factor for OS.

**Figure 4:**
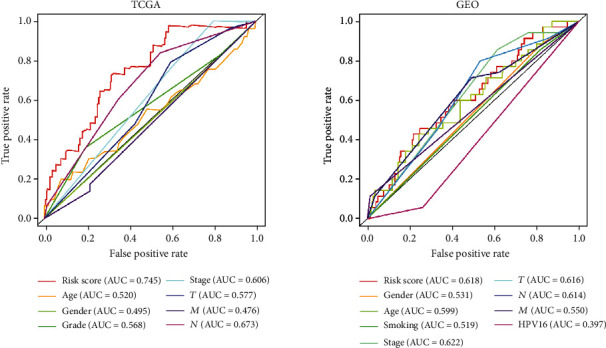
The predictor of clinicopathological in HNSCC was also analyzed, and we found the AUC for age, gender, grade, stage, T, M, and N was 0.520, 0.495, 0.568, 0.606, 0.577, 0.476, and 0.673, respectively, in TCGA, and the AUC for age, gender, stage, T, M, N, smoking, and HPV16-pos was 0.599, 0.531, 0.622, 0.606, 0.616, 0.550, 0.614, 0.519, and 0.397, respectively, in GEO.

Our OS analysis demonstrated that, unlike the low-risk group, the high-risk HNSCC group was associated with a worse prognosis (*P* < 0.01) (Figure 1). We showed that the mortality rate was higher in the high-risk HNSCC patients, and the increase in the patients' risk score was proportional to the death rate (Figure 2). Next, we used the univariate and multivariate COX analyses to determine the risk factors which defined the prognostic model based on thirty metabolic-related genes. We demonstrate that the 30 metabolic-related gene signatures could robustly and independently predict prognosis and OS (Figure 3). On the other hand, we evaluated whether the metabolic-related gene patterns could serve as an early predictor of incidence in HNSCC. The ROC curve and the model demonstrated an AUC of 0.745 in the TCGA and 0.618 in the GEO datasets. Taken together, these data indicated that the constructed prognostic tool has moderate sensitivity and specificity. In addition, analysis of the clinicopathological factors in HNSCC showed that the AUC for age, gender, grade, stage, T, M, and N was 0.520, 0.495, 0.568, 0.606, 0.577, 0.476, and 0.673, respectively, in the TCGA dataset, and the AUC for age, gender, stage, T, M, N, smoking, and HPV16-pos was 0.599, 0.531, 0.622, 0.606, 0.616, 0.550, 0.614, 0.519, and 0.397, respectively, in the GEO dataset (Figure 4).

### 3.3. Construction and Validation of the Predictive Nomogram in the TCGA and GEO Cohorts

**Figure 5:**
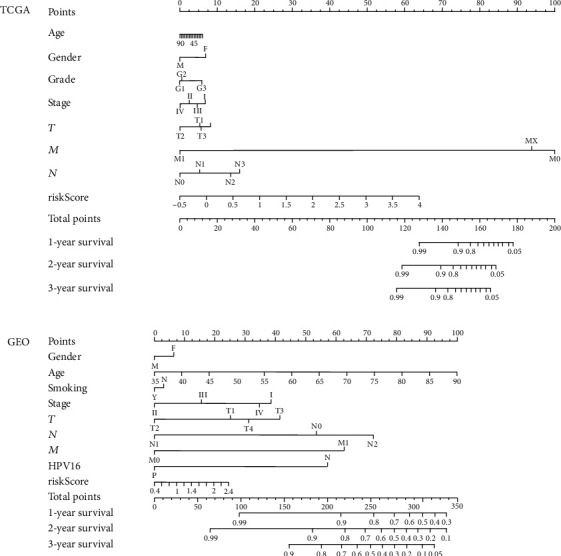
The model ultimately included 7 features in TCGA: age, gender, grade, stage, T, M, and N and 8 features in TCGA: age, gender, stage, T, M, N, smoking, and HPV16-pos.

The nomogram was constructed from the clinicopathological data as well as the developed prognostic model. Through the LASSO logistic regression algorithm, the most important prediction markers were selected in the training data set, which reflected the final prediction model. The model included 7 features in TCGA: age, gender, grade, stage, T, M, and N as well as 8 features in GEO: age, gender, stage, T, M, N, smoking, and HPV16-pos (Figure 5). Integrating our prognostic model with clinicopathological analysis fortified the forecasting sensitivity and specificity for 1-, 2-, and 3-year OS, thus, increasing the usefulness in the clinical management of patients.

### 3.4. Gene Set Enrichment Analyses

**Figure 6:**
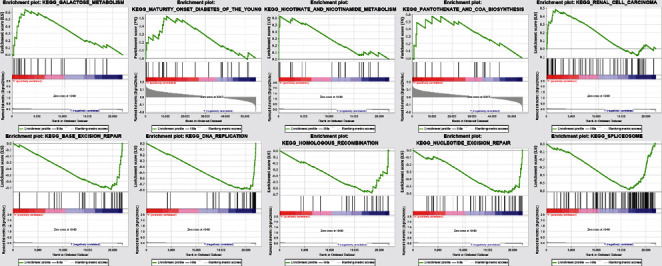
Then samples were divided into high- and low-risk groups as training set to distinguish the potential function and elucidate the significant survival difference utilizing GSEA.

Here, we split the samples into high- and low-risk groups to distinguish the potential functions and elucidate the significant survival differences in the GSEA. Annotated gene set c2.cp.kegg.v6.0.symbols.gmt was selected as the reference gene sets, which included terms with NOM < 0.05. Gene set permutations were executed multiple times for every examination. A great majority of the metabolic-related pathways such as galactose metabolism, nicotinate/nicotinamide, and pantothenate/COA biosynthesis or metabolic disease-related perturbations were enriched in the high-risk group. On the other hand, most of the nonmetabolic-related pathways such as base excision repair, spliceosome, homologous recombination, nucleotide excision, and DNA replication were enriched in the low-risk group (Figure 6 and Table 3).

### 3.5. Online Database Analysis

**Figure 7:**
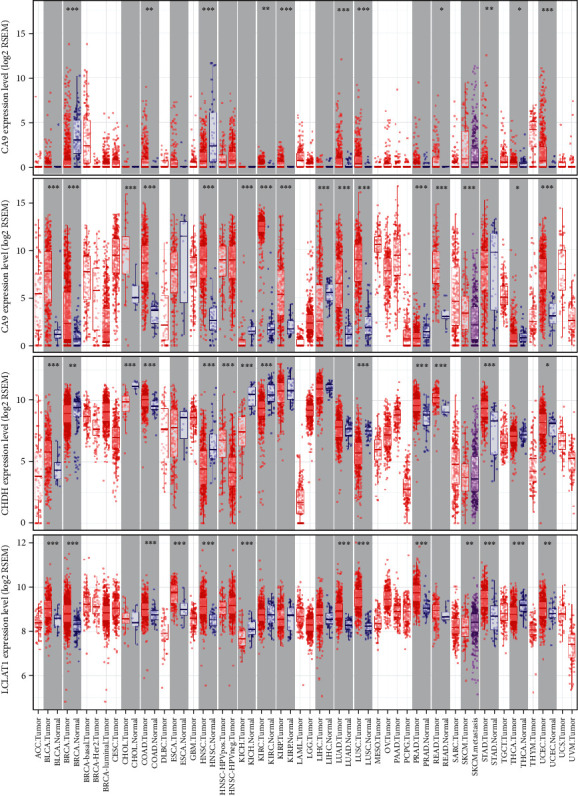
Differential expression of CA6, CA9, LCLAT1, and CHDH between tumors and normal tissues based on TIMER database.

**Figure 8:**
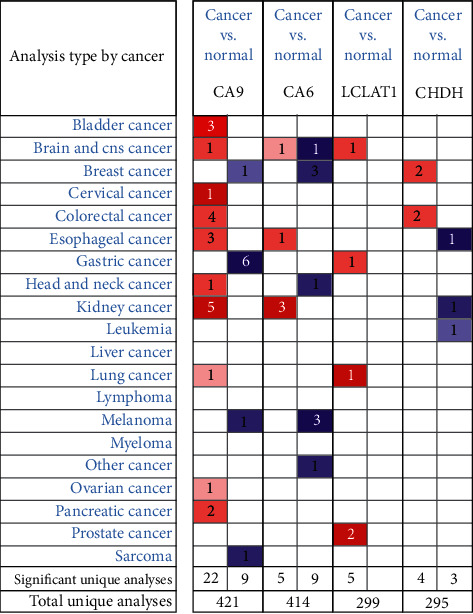
The transcriptional levels of *CA6*, *CA9*, *LCLAT1*, and *CHDH* in cancers and normal samples. Redder means higher expression and bluer means lower expression. (ONCOMINE Database).

**Figure 9:**
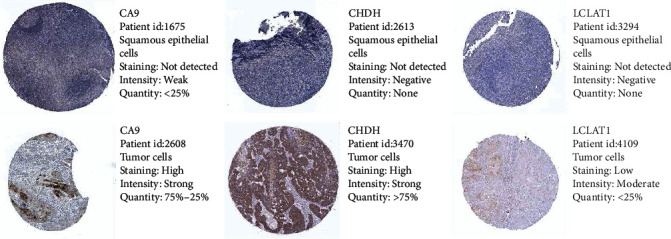
Representative protein expressions of *CA9*, *LCLAT1*, and *CHDH* were explored in the HPA database.

**Figure 10:**
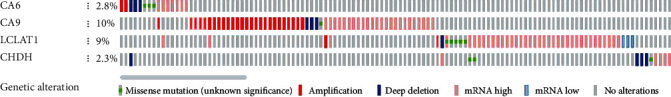
*CA6*, *CA9*, *LCLAT1*, and *CHDH* gene expression and mutation analysis.

To provide new insights into the potential functions, expression patterns, molecular mechanisms, and distinct prognostic value, we used multidimensional survey techniques to explore *CA6*, *CA9*, *LCLAT1*, and *CHDH* based on variations in the copy numbers or gene expression profile in the HNSCC patients. In agreement with our findings, data from both the TIMER database and Oncomine showed that *CA6* was significantly downregulated, while *CA9* was significantly overexpressed in HNSCC patients (Figures 7 and 8). Despite the limited data in the Oncomine, the *LCLAT1* mRNA expression was overexpressed while the *CHDH* expression was downregulated in HNSCC in the TIMER database. Representative protein expression levels for *CA9*, *LCLAT1*, and *CHDH* were explored in the HPA database as shown in Figure 9. We showed that *CA9* has the most frequent genetic variations (10%), and the most pronounced changes were amplification of mutations (Figure 10). In summary, we verified the abnormal expression profiles for these genes in HNSCC, and the genetic changes might explain the abnormal expression.

## 4. Discussion

Proliferating cancer cells must maintain sufficient energy and a library of metabolic intermediates to build the macromolecules required for growth. The molecules include DNA, proteins, and lipids [16]. Because the metabolic profile could distinguish the tumor cells from the normal cells, metabolic signaling pathways have become ideal targets for therapeutic intervention for cancer patients. In this study, we identified a novel and effective metabolic-related prognostic gene signature based on the TCGA dataset and validated it in the GSE65858 dataset. Our constructed signature had a strong prognostic value and may represent the metabolic status of patients with HNSCC. Therefore, the signature could be used as a potential biomarker and therapeutic target in the metabolic signaling pathways.

In our study, we downloaded transcriptome data from the TCGA database and verified it using the GEO dataset as well as the metabolic-related genes extract from the GSEA metabolic signaling pathways. We first evaluated the relationship between the differentially expressed RNA, immune-related genes, and transcription factors in HNSCC patients. Univariate Cox regression model found 51 survival-related genes, whereby *LCLAT1* was associated with the highest risk while *CHDH* denoted the least risk. Cardiolipin (CL) types of polyunsaturated fatty acids, especially DHA (C22: 6n3), increased in ALCAT1-expressing cells, while C16-C18 fatty acids significantly decreased [17]. A recent study showed that *ALCAT1* is critical for coupling mitochondrial respiration and metabolic plasticity [18]. Wang et al. reported that forced expression of *ALCAT1* in primary hepatocytes led to multiple defects including steatosis, defective autophagy, and mitochondrial dysfunction [19]. Meanwhile, *ALCAT1* can promote ROS production and is critical for coupling mitochondrial respiration and metabolic plasticity [20]. However, there was limited data on the role of *ALCAT1* in tumors. Here, we hypothesize that the *ALCAT1* might play a regulatory role in cardiolipin remodeling in response to oxidative stress and stimulate mitochondrial activity in HNSCC cancers. Choline dehydrogenase (*CHDH*) localizes to the mitochondrion, and variations in this gene can affect susceptibility to choline deficiency. *CHDH* strongly predicted clinical outcome in breast cancer patients receiving tamoxifen monotherapy [21]. Choline is an essential nutrient required for methyl group metabolism, and *CHDH* is associated with an increased risk of breast cancer [22]. There is no available data on the role of *CHDH* in HNSCC.

By the use of ROC curves, we next interrogated whether the metabolic-related gene patterns could serve as an early predictor for the incidence of HNSCC. Our model demonstrated an AUC of 0.745 and 0.618 in the TCGA and GEO datasets. Integrating our prognostic model with the clinicopathological indicators enhanced the prediction sensitivity and specificity for the 1-, 2-, and 3-year OS, thus, better clinical management. Our further analysis of the survival difference using the GSEA showed that the majority of the metabolic-related pathways such as galactose and nicotinamide metabolism were enriched in the high-risk group while most of the nonmetabolic-related pathways were enriched in the low-risk group. Galactose is an essential molecule and plays a pivotal role in energy transfer and galactosylation of complex molecules. On the other hand, nicotinamide adenine dinucleotide (NAD) plays a central role in energy metabolism and integrates cell metabolism with signaling and gene expression [23]. NAD biosynthesis is dependent on nicotinamide/nicotinate single-nucleotide adenylate transferase [24]. Therefore, high-risk patients may benefit from metabolic therapy, while low-risk patients may benefit from nonmetabolism-targeted therapy. However, there is a need for more studies on the relationship between gene signatures, metabolic microenvironment, and metabolic therapies. Our data provide a promising direction in elucidating the underlying molecular mechanisms for the interrogated signatures. In conclusion, our signatures may reflect metabolic microenvironment disorders and provide potential biomarkers for metabolic therapy and prediction of prognosis after treatment.

Our analysis of the 327 metabolic genes in the TCGA database showed that the *CA9* had the largest logFC in the upregulated category, while *CA6* was downregulated. Carbonic anhydrases (*CAs*) are a large class of zinc metal enzymes that catalyze the reversible hydration of carbon dioxide. They are involved in various biological processes, including bone resorption, respiration, calcification, and acid-base balance. A previous study showed that pancreatic ductal adenocarcinoma (PDACs) cells that express an activated KRAS increase the expression of *CA9*, via stabilization of hypoxia-inducible factor 1 subunit alpha (*HIF1A*) and *HIF2A*, which eventually regulates the pH and glycolysis [25]. Similarly, *CA9* is an independent prognostic factor for OSCC patients and therefore a potential therapeutic target [26]. *CA6* encodes several isoenzymes which are only found in salivary glands. Saliva and proteins may play a role in the reversible hydration of carbon dioxide. *CA6* is a specific marker for salivary gland serous acinar cells and acinar cell carcinoma (AciCC). *CA6* has the same sensitivity and specificity as *DOG1* in the differential diagnosis of AciCC and breast analogs (MASC) [27]. However, data on the role of *CA9* and *CA6* in the prognosis of human HNSCC remains scant. Our study identified a novel metabolic gene signature for the prediction of HNSCC prognosis based on the TCGA data set. Our signatures might reflect the disorders in the metabolic microenvironment and provide potential biomarkers for metabolic therapy and monitoring of the treatment response. However, the metabolic gene signatures for prediction must be verified in more independent cohorts and functional experiments. It is, however, important to mention that our study was limited by the relatively small sample size and the fact that our results were not verified in clinical samples.

## 5. Conclusion

Taken together, our data defined a novel metabolic gene signature for the prediction of HNSCC prognosis based on the TCGA dataset. Our signatures reflect the metabolic microenvironment disorders and provide useful biomarkers for metabolic therapy and prediction of the response to the treatment.

## Figures and Tables

**Table 1:** Prognostic related metabolic genes, HR > 1 is a high-risk gene, and HR < 1 is a low-risk gene.

Id	HR	HR.95L	HR.95H	*P* value
HEXB	1.038	1.006	1.071	0.019
ACAT1	1.087	1.034	1.143	0.001
P4HA1	1.019	1.007	1.030	0.001
GNPDA1	1.065	1.013	1.119	0.013
POLE2	0.882	0.791	0.982	0.023
ACACB	0.649	0.445	0.945	0.024
SMS	1.007	1.002	1.011	0.001
AGPS	1.053	1.010	1.097	0.014
PYGL	1.009	1.003	1.014	0.003
ACAA1	0.890	0.802	0.988	0.029
MTHFD2	1.035	1.017	1.052	4.745
PLCB3	1.027	1.008	1.045	0.005
POLE	0.857	0.764	0.960	0.008
POLD2	1.013	1.003	1.022	0.006
MINPP1	1.082	1.015	1.153	0.016
KYNU	1.074	1.014	1.136	0.013
PIK3C2B	0.825	0.729	0.932	0.002
CHDH	0.580	0.400	0.839	0.003
G6PD	1.003	1.000	1.006	0.033
POLD1	0.959	0.924	0.994	0.025
PTDSS1	1.016	1.001	1.030	0.031
ENTPD1	0.813	0.710	0.930	0.002
LCLAT1	1.144	1.044	1.251	0.003
PFKP	1.012	1.001	1.021	0.024
PIP4K2A	0.927	0.859	0.998	0.046
DNMT1	0.959	0.932	0.986	0.003
ADK	1.040	1.017	1.061	0.001
NEU1	1.033	1.001	1.066	0.041
GATM	0.897	0.827	0.972	0.008
TXNDC12	1.022	1.000	1.043	0.046
ADA	1.036	1.019	1.053	2.829e-05
PAFAH1B2	1.043	1.013	1.074	0.004
PLA2G2D	0.864	0.778	0.958	0.005
DGKQ	0.908	0.845	0.974	0.007
NAGK	0.922	0.869	0.976	0.005
FTH1	1.002	1.000	1.002	0.010
ADH7	1.006	1.000	1.010	0.030
ACOX3	0.897	0.830	0.969	0.005
SHMT1	0.923	0.861	0.988	0.022
ASNS	1.030	1.011	1.049	0.001
HPRT1	1.029	1.016	1.041	5.217e-06
ATIC	1.038	1.016	1.061	0.001
LDHA	1.002	1.000	1.003	0.007
PRPS1	1.035	1.014	1.055	0.001
NADSYN1	1.037	1.001	1.074	0.040
GSTO1	1.003	1.000	1.006	0.049
TXNRD1	1.005	1.000	1.010	0.034
RDH11	1.029	1.006	1.052	0.012
PAICS	1.024	1.005	1.043	0.010

**Table 2:** Then Lasso-penalized Cox analysis found 30 genes to build the prognostic model.

Gene	Coef	Gene	Coef
*HEXB*	0.01247	*DNMT1*	-0.01837
*ACAT1*	0.01996	*ADK*	0.00435
*GNPDA1*	0.02995	*ADA*	0.01480
*POLE2*	-0.17051	*PAFAH1B2*	0.00517
*SMS*	0.00156	*PLA2G2D*	-0.03567
*AGPS*	0.00050	*DGKQ*	-0.03222
*PYGL*	0.00442	*ADH7*	0.00527
*ACAA1*	-0.00201	*ACOX3*	-0.00481
*MTHFD2*	0.00689	*ASNS*	0.00862
*PLCB3*	0.02056	*HPRT1*	0.00982
*POLD2*	0.00170	*ATIC*	0.00835
*KYNU*	0.03766	*PRPS1*	0.01487
*ENTPD1*	-0.01556	*NADSYN1*	0.01067
*LCLAT1*	0.02843	*RDH11*	0.00258
*PIP4K2A*	-0.00409	*HADHB*	0.01222

**Table 3:** Gene sets enriched in phenotype high and low.

Gene set name	Size	NES	NOM *P* value
KEGG_GALACTOSE_METABOLISM	23	1.62	0.010
KEGG_NICOTINATE_AND_NICOTINAMIDE_METABOLISM	19	1.45	0.034
KEGG_RENAL_CELL_CARCINOMA	64	1.41	0.046
KEGG_PANTOTHENATE_AND_COA_BIOSYNTHESIS	16	1.59	0.031
KEGG_MATURITY_ONSET_DIABETES_OF_THE_YOUNG	25	1.53	0.033
KEGG_BASE_EXCISION_REPAIR	33	-1.88	0.000
KEGG_SPLICEOSOME	109	-1.82	0.002
KEGG_HOMOLOGOUS_RECOMBINATION	24	-1.65	0.020
KEGG_NUCLEOTIDE_EXCISION_REPAIR	44	-1.64	0.022
KEGG_DNA_REPLICATION	36	-1.63	0.013

NES: normalized enrichment score; NOM: nominal; Gene sets with NOM *P* value < 0.05 are considered as significant.

## Data Availability

Data sharing is not applicable to this article as no datasets were generated or analyzed during the current study.

## Abbreviations

HNSCC: Head and neck squamous cell carcinoma
TCGA: The Cancer Genome Atlas
HPV: Human papillomavirus
ROC: Receiver operating characteristic
GSEA: Gene set enrichment analysis
GEO: Gene Expression Omnibus
OS: Overall survival.

## Supplementary Materials

Supplementary Materials Supplementary Table 1: the expression of included consistent altered metabolic genes in TCGA-HNSCC.
