# Supplementary material for: A Metabolic Gene Signature to Predict Overall Survival in Head and Neck Squamous Cell Carcinoma
Source: Mediators Inflamm. 2020 Dec 30;2020:6716908. doi: 10.1155/2020/6716908 (PMC7787728; doi:10.1155/2020/6716908)
Supplement: Supplementary Materials — Supplementary Table 1: the expression of included consistent altered metabolic genes in TCGA-HNSCC. [file 6716908.f1.docx]

**A metabolic gene signature to predict overall survival in Head and neck squamous cell carcinoma**

**Zeng-Hong Wu, MD; Yun Tang, MD; Yue Zhou, MD.**

Supplementary Table 1. The expression of included consistent altered metabolic genes in TCGA-HNSCC.

| gene | conMean | treatMean | logFC | pValue | fdr | Expression change |
| --- | --- | --- | --- | --- | --- | --- |
| CA9 | 0.044644995 | 16.44067937 | 8.524555752 | 3.01E-16 | 4.13E-15 | Up-regulated |
| TK1 | 5.178303007 | 48.40610777 | 3.224637803 | 9.86E-24 | 7.02E-21 | Up-regulated |
| ODC1 | 9.935377812 | 92.26677062 | 3.215164428 | 6.98E-09 | 2.39E-08 | Up-regulated |
| WARS | 8.516562761 | 70.05339165 | 3.040111712 | 1.38E-13 | 1.08E-12 | Up-regulated |
| IDO1 | 2.053870627 | 12.80382241 | 2.640157357 | 1.96E-08 | 6.16E-08 | Up-regulated |
| CHST11 | 1.21703818 | 6.404216052 | 2.395647551 | 2.61E-20 | 1.86E-18 | Up-regulated |
| LPCAT1 | 2.184780261 | 10.03075025 | 2.198869426 | 3.33E-21 | 2.97E-19 | Up-regulated |
| P4HA1 | 3.503826149 | 15.78428198 | 2.171485535 | 4.13E-19 | 1.58E-17 | Up-regulated |
| NT5E | 2.087309916 | 9.302022528 | 2.155899611 | 3.43E-08 | 1.04E-07 | Up-regulated |
| TYMP | 21.36408637 | 95.00324166 | 2.152789119 | 7.47E-14 | 6.13E-13 | Up-regulated |
| ADA | 2.012759223 | 8.283899348 | 2.041135425 | 5.26E-19 | 1.78E-17 | Up-regulated |
| PTGS2 | 2.05913407 | 8.458734629 | 2.038404095 | 0.001161667 | 0.001817818 | Up-regulated |
| CMPK2 | 1.284417082 | 4.908830088 | 1.934265474 | 1.51E-11 | 8.10E-11 | Up-regulated |
| TYMS | 3.139422412 | 11.75299563 | 1.904457458 | 5.33E-15 | 5.58E-14 | Up-regulated |
| CA2 | 8.963755204 | 32.6922848 | 1.866775053 | 0.000340827 | 0.00056831 | Up-regulated |
| ACP5 | 6.833810982 | 24.24345952 | 1.826833335 | 1.60E-15 | 1.81E-14 | Up-regulated |
| P4HA2 | 2.723729284 | 9.638679106 | 1.823252133 | 3.69E-18 | 9.74E-17 | Up-regulated |
| IMPDH1 | 5.935818164 | 19.24314214 | 1.696825588 | 2.41E-17 | 4.90E-16 | Up-regulated |
| PLA2G7 | 1.27918594 | 4.125716239 | 1.689418611 | 3.36E-15 | 3.57E-14 | Up-regulated |
| IL4I1 | 1.307667274 | 4.127151052 | 1.658150737 | 3.53E-12 | 2.13E-11 | Up-regulated |
| RRM2 | 4.775620683 | 14.88600243 | 1.640196221 | 1.94E-14 | 1.79E-13 | Up-regulated |
| SMS | 9.544289749 | 29.52810477 | 1.629379015 | 3.16E-18 | 8.64E-17 | Up-regulated |
| SPHK1 | 4.384433982 | 12.99660469 | 1.567672262 | 1.05E-14 | 1.04E-13 | Up-regulated |
| CHIT1 | 0.833025828 | 2.460121749 | 1.562296583 | 4.24E-09 | 1.51E-08 | Up-regulated |
| TXNRD1 | 5.3385769 | 15.42566533 | 1.530805596 | 7.00E-06 | 1.50E-05 | Up-regulated |
| HPRT1 | 7.068535939 | 20.35070428 | 1.525595388 | 1.78E-21 | 1.81E-19 | Up-regulated |
| PYGL | 10.63875509 | 30.48827542 | 1.518925204 | 6.33E-10 | 2.64E-09 | Up-regulated |
| MTHFD1L | 2.615413953 | 7.347085434 | 1.490132745 | 2.16E-17 | 4.66E-16 | Up-regulated |
| DTYMK | 4.10295081 | 11.18010508 | 1.446199985 | 1.01E-16 | 1.60E-15 | Up-regulated |
| DNMT3B | 1.006815705 | 2.734624509 | 1.441543124 | 4.06E-22 | 7.24E-20 | Up-regulated |
| ASNS | 2.518183121 | 6.790231974 | 1.431077663 | 3.74E-12 | 2.24E-11 | Up-regulated |
| POLR2H | 5.022373297 | 13.5091764 | 1.427498553 | 5.95E-16 | 7.31E-15 | Up-regulated |
| POLD1 | 2.919495587 | 7.773172302 | 1.412784365 | 6.60E-19 | 2.14E-17 | Up-regulated |
| DNMT1 | 3.763830769 | 9.903619618 | 1.395754142 | 4.10E-16 | 5.31E-15 | Up-regulated |
| MIF | 15.20777608 | 39.50874848 | 1.377362951 | 1.38E-12 | 9.02E-12 | Up-regulated |
| PIK3CD | 2.020959615 | 5.182663936 | 1.358653354 | 1.87E-16 | 2.76E-15 | Up-regulated |
| GMPS | 4.147206841 | 10.39964485 | 1.326322352 | 1.29E-18 | 3.67E-17 | Up-regulated |
| GLS | 2.84830808 | 6.889043982 | 1.274198588 | 1.90E-16 | 2.76E-15 | Up-regulated |
| PTDSS1 | 6.345803426 | 15.29865419 | 1.269530009 | 2.43E-19 | 1.05E-17 | Up-regulated |
| MTHFD2 | 5.34738671 | 12.68972099 | 1.246754431 | 1.92E-15 | 2.11E-14 | Up-regulated |
| PSPH | 3.114267033 | 7.323304319 | 1.233602095 | 2.75E-08 | 8.47E-08 | Up-regulated |
| PRIM2 | 1.523039963 | 3.558280959 | 1.224226632 | 1.60E-22 | 5.71E-20 | Up-regulated |
| PDE7A | 1.963015195 | 4.566562373 | 1.218037198 | 3.11E-14 | 2.73E-13 | Up-regulated |
| PAFAH1B3 | 6.996154773 | 16.24064566 | 1.214974879 | 9.13E-09 | 3.07E-08 | Up-regulated |
| SHMT2 | 9.863886504 | 22.52605319 | 1.191366455 | 7.78E-16 | 9.39E-15 | Up-regulated |
| GAA | 7.918566746 | 18.01681976 | 1.186033144 | 1.47E-08 | 4.69E-08 | Up-regulated |
| GPX7 | 2.238218975 | 5.045935631 | 1.172770614 | 7.16E-07 | 1.80E-06 | Up-regulated |
| SULT1E1 | 1.156033187 | 2.603754427 | 1.171410572 | 0.002251563 | 0.003403637 | Up-regulated |
| GLA | 4.544232812 | 10.23503607 | 1.171407532 | 3.27E-17 | 6.27E-16 | Up-regulated |
| POLA2 | 2.155086095 | 4.820414657 | 1.161411748 | 2.15E-19 | 1.05E-17 | Up-regulated |
| GSTO1 | 22.84378131 | 50.76041409 | 1.15190236 | 5.43E-14 | 4.61E-13 | Up-regulated |
| RDH16 | 1.136500232 | 2.521724467 | 1.149812671 | 6.97E-12 | 4.00E-11 | Up-regulated |
| G6PD | 13.30366139 | 29.43715401 | 1.145814844 | 0.003754867 | 0.005467209 | Up-regulated |
| TXNDC12 | 7.463443373 | 16.3422471 | 1.130693073 | 6.54E-20 | 3.88E-18 | Up-regulated |
| ISYNA1 | 3.51445509 | 7.667418626 | 1.125439931 | 0.002921207 | 0.004324114 | Up-regulated |
| DGUOK | 9.573951561 | 20.53819258 | 1.101122814 | 2.72E-16 | 3.80E-15 | Up-regulated |
| POLR2K | 8.150509393 | 17.46502465 | 1.099506546 | 1.60E-17 | 3.57E-16 | Up-regulated |
| PYCR1 | 5.825082572 | 12.43741464 | 1.094336219 | 6.39E-06 | 1.38E-05 | Up-regulated |
| CYP26B1 | 2.251057395 | 4.805702092 | 1.094144376 | 0.000299657 | 0.000509203 | Up-regulated |
| NME1 | 6.76663955 | 14.38821128 | 1.088375804 | 2.61E-12 | 1.62E-11 | Up-regulated |
| LDHA | 63.04990355 | 132.8523288 | 1.075257448 | 6.33E-11 | 3.09E-10 | Up-regulated |
| MINPP1 | 2.500444306 | 5.256499483 | 1.071917899 | 3.16E-16 | 4.25E-15 | Up-regulated |
| CAD | 4.010000476 | 8.40014158 | 1.066811236 | 1.65E-13 | 1.23E-12 | Up-regulated |
| PYCR2 | 6.298428091 | 13.13471646 | 1.060321335 | 2.31E-13 | 1.69E-12 | Up-regulated |
| KYNU | 1.269848701 | 2.638934628 | 1.055298998 | 4.26E-07 | 1.11E-06 | Up-regulated |
| SGPP1 | 2.28999661 | 4.756995087 | 1.054705073 | 5.80E-11 | 2.91E-10 | Up-regulated |
| PRPS1 | 6.363348696 | 13.20415004 | 1.053133352 | 6.94E-17 | 1.18E-15 | Up-regulated |
| PSAT1 | 9.068002773 | 18.81031884 | 1.052667565 | 1.78E-05 | 3.63E-05 | Up-regulated |
| PRIM1 | 2.023085293 | 4.157967021 | 1.039321171 | 1.26E-14 | 1.20E-13 | Up-regulated |
| HK3 | 1.107383418 | 2.274457075 | 1.038367384 | 6.22E-11 | 3.05E-10 | Up-regulated |
| ATIC | 7.381381445 | 15.13917307 | 1.036323654 | 2.32E-17 | 4.87E-16 | Up-regulated |
| NAGS | 1.288265716 | 2.632005613 | 1.030732373 | 1.23E-15 | 1.43E-14 | Up-regulated |
| CKB | 13.8642323 | 28.19430933 | 1.024036269 | 0.021847873 | 0.027489571 | Up-regulated |
| ALDOC | 4.935633066 | 10.01658603 | 1.021083827 | 0.001093635 | 0.001715127 | Up-regulated |
| DUT | 5.60938306 | 11.31421756 | 1.012222806 | 4.06E-12 | 2.37E-11 | Up-regulated |
| GCLC | 4.992471472 | 10.02610008 | 1.005934451 | 0.001738429 | 0.002661853 | Up-regulated |
| GGT5 | 2.286617228 | 4.586461393 | 1.004166612 | 2.69E-09 | 1.00E-08 | Up-regulated |
| ADCY3 | 3.566181055 | 7.128903839 | 0.99930031 | 2.48E-12 | 1.55E-11 | Up-regulated |
| POLR2G | 10.85241337 | 21.68115195 | 0.998425504 | 1.05E-15 | 1.25E-14 | Up-regulated |
| LAP3 | 12.71916204 | 25.37002113 | 0.996121095 | 4.89E-05 | 9.49E-05 | Up-regulated |
| PAICS | 7.490271278 | 14.91218521 | 0.993401808 | 1.88E-12 | 1.21E-11 | Up-regulated |
| LDHB | 39.17496523 | 77.57990302 | 0.985750978 | 9.66E-06 | 2.03E-05 | Up-regulated |
| PLCG1 | 2.914705206 | 5.750056016 | 0.980226035 | 8.52E-19 | 2.53E-17 | Up-regulated |
| TPI1 | 103.8495308 | 203.9190528 | 0.97350188 | 3.16E-14 | 2.74E-13 | Up-regulated |
| FTH1 | 76.64645424 | 149.7549715 | 0.966312941 | 3.64E-09 | 1.31E-08 | Up-regulated |
| GNPDA1 | 4.409427859 | 8.612691468 | 0.965872678 | 1.45E-19 | 7.92E-18 | Up-regulated |
| RRM1 | 11.46399785 | 22.32824641 | 0.961759706 | 8.82E-12 | 4.94E-11 | Up-regulated |
| POLE2 | 1.545946092 | 3.009363098 | 0.960968174 | 5.32E-16 | 6.65E-15 | Up-regulated |
| B4GALT2 | 8.832014396 | 17.17387284 | 0.959400986 | 8.44E-17 | 1.37E-15 | Up-regulated |
| POLE3 | 14.35732255 | 27.83450743 | 0.955087822 | 1.35E-14 | 1.27E-13 | Up-regulated |
| ADH5 | 9.967570005 | 19.32242494 | 0.954962424 | 2.80E-14 | 2.55E-13 | Up-regulated |
| PCYT1A | 6.450629742 | 12.47863205 | 0.951947874 | 2.94E-14 | 2.62E-13 | Up-regulated |
| MBOAT7 | 5.803759634 | 11.19577101 | 0.947894208 | 2.68E-15 | 2.89E-14 | Up-regulated |
| SMPD4 | 4.281603739 | 8.072380472 | 0.914842893 | 7.86E-18 | 1.93E-16 | Up-regulated |
| CD38 | 1.527679929 | 2.864635587 | 0.907009315 | 1.62E-05 | 3.34E-05 | Up-regulated |
| CPOX | 3.584066225 | 6.715868568 | 0.905976701 | 7.81E-13 | 5.45E-12 | Up-regulated |
| GLO1 | 24.02207688 | 44.9908802 | 0.905271704 | 2.74E-13 | 1.99E-12 | Up-regulated |
| GGT1 | 1.563367322 | 2.906600611 | 0.89467606 | 1.75E-05 | 3.60E-05 | Up-regulated |
| ACYP1 | 1.978887852 | 3.669234703 | 0.890789336 | 2.98E-11 | 1.55E-10 | Up-regulated |
| NPL | 2.432014685 | 4.504489409 | 0.889211643 | 3.34E-06 | 7.34E-06 | Up-regulated |
| RENBP | 1.853690677 | 3.430429902 | 0.887988863 | 2.95E-08 | 9.03E-08 | Up-regulated |
| UXS1 | 2.992125844 | 5.496248755 | 0.877276447 | 1.63E-13 | 1.23E-12 | Up-regulated |
| UPP1 | 10.4968816 | 19.27455124 | 0.876736473 | 0.000121471 | 0.000222906 | Up-regulated |
| UMPS | 3.28304985 | 6.020222658 | 0.87478019 | 4.41E-14 | 3.78E-13 | Up-regulated |
| GFPT2 | 2.198304258 | 4.010355757 | 0.867339146 | 8.10E-07 | 2.02E-06 | Up-regulated |
| GSTP1 | 269.8850549 | 491.4152377 | 0.864597504 | 9.45E-06 | 2.00E-05 | Up-regulated |
| GNPNAT1 | 3.850560076 | 6.945212524 | 0.850950536 | 7.13E-12 | 4.06E-11 | Up-regulated |
| ITPK1 | 4.376815074 | 7.86360105 | 0.845308701 | 8.31E-13 | 5.74E-12 | Up-regulated |
| PC | 5.305506259 | 9.523495061 | 0.844000709 | 0.000339696 | 0.000567755 | Up-regulated |
| LPCAT2 | 2.682907142 | 4.759909534 | 0.827137032 | 5.67E-09 | 1.97E-08 | Up-regulated |
| SRM | 19.0434435 | 33.59452094 | 0.818931582 | 1.92E-10 | 8.63E-10 | Up-regulated |
| NUDT5 | 5.187760177 | 9.108357876 | 0.81207919 | 2.27E-11 | 1.19E-10 | Up-regulated |
| PPAT | 2.098639554 | 3.682782465 | 0.81134178 | 2.19E-13 | 1.62E-12 | Up-regulated |
| FLAD1 | 5.215687496 | 9.133380166 | 0.808291454 | 1.15E-14 | 1.12E-13 | Up-regulated |
| POLE | 1.889413468 | 3.300952322 | 0.804943854 | 1.01E-14 | 1.01E-13 | Up-regulated |
| GSS | 16.67054209 | 29.12345823 | 0.804880658 | 7.84E-11 | 3.80E-10 | Up-regulated |
| PIP4K2C | 9.77105569 | 17.06548688 | 0.804495227 | 1.08E-12 | 7.23E-12 | Up-regulated |
| NEU1 | 5.349903497 | 9.300117214 | 0.797736031 | 1.66E-11 | 8.83E-11 | Up-regulated |
| POLE4 | 7.283478127 | 12.63837531 | 0.795111554 | 4.22E-11 | 2.14E-10 | Up-regulated |
| PFKFB4 | 1.741488535 | 2.986745243 | 0.778253212 | 1.34E-11 | 7.32E-11 | Up-regulated |
| POLD2 | 16.51282748 | 28.25052246 | 0.774690375 | 1.66E-10 | 7.52E-10 | Up-regulated |
| POLR3K | 3.895100644 | 6.644333756 | 0.770463938 | 2.14E-10 | 9.45E-10 | Up-regulated |
| PIK3CA | 2.3194931 | 3.92201641 | 0.757786017 | 2.09E-11 | 1.10E-10 | Up-regulated |
| ITPKA | 1.174230432 | 1.982226542 | 0.755406301 | 3.47E-10 | 1.49E-09 | Up-regulated |
| NME4 | 5.525863283 | 9.279558587 | 0.747856312 | 1.59E-05 | 3.30E-05 | Up-regulated |
| GART | 5.573271137 | 9.287960951 | 0.736837565 | 6.18E-11 | 3.05E-10 | Up-regulated |
| LCLAT1 | 1.93450952 | 3.213480976 | 0.732169104 | 1.70E-16 | 2.63E-15 | Up-regulated |
| B4GALT1 | 21.95390248 | 36.37552198 | 0.728490537 | 6.68E-06 | 1.43E-05 | Up-regulated |
| PGP | 3.513831094 | 5.81199539 | 0.725988714 | 1.27E-10 | 5.98E-10 | Up-regulated |
| PLA2G2D | 1.149401732 | 1.897950412 | 0.723559171 | 0.002356285 | 0.003546881 | Up-regulated |
| DCK | 3.873310461 | 6.392309512 | 0.722770121 | 2.21E-08 | 6.93E-08 | Up-regulated |
| PLCB3 | 9.146417666 | 15.06720455 | 0.72013307 | 1.97E-06 | 4.59E-06 | Up-regulated |
| SEPHS2 | 11.22477147 | 18.41724192 | 0.714370953 | 3.35E-10 | 1.45E-09 | Up-regulated |
| GGCT | 13.73476844 | 22.52613318 | 0.713767095 | 6.32E-07 | 1.61E-06 | Up-regulated |
| LRAT | 0.977612077 | 1.595554029 | 0.70672345 | 0.018619226 | 0.023757865 | Up-regulated |
| LPGAT1 | 3.282062985 | 5.351279832 | 0.705281048 | 8.35E-11 | 4.02E-10 | Up-regulated |
| AKR1B1 | 9.622562934 | 15.68447711 | 0.704844327 | 1.52E-06 | 3.59E-06 | Up-regulated |
| MTHFD1 | 5.222523003 | 8.500290137 | 0.702765143 | 1.07E-08 | 3.51E-08 | Up-regulated |
| UGDH | 8.874689688 | 14.43628959 | 0.701931409 | 0.02624314 | 0.03289633 | Up-regulated |
| HK2 | 14.02783793 | 22.81780222 | 0.701867173 | 0.001750847 | 0.002675114 | Up-regulated |
| TH | 0.974985447 | 1.584206875 | 0.700308154 | 0.000109158 | 0.000201872 | Up-regulated |
| SDS | 1.659612986 | 2.692219878 | 0.697949391 | 2.50E-10 | 1.09E-09 | Up-regulated |
| NT5C | 8.446187487 | 13.69789143 | 0.697581653 | 2.00E-07 | 5.66E-07 | Up-regulated |
| HEXB | 7.691910089 | 12.46492427 | 0.696460314 | 7.74E-10 | 3.13E-09 | Up-regulated |
| PNPT1 | 3.946527017 | 6.383682091 | 0.693805181 | 1.06E-10 | 5.02E-10 | Up-regulated |
| PGS1 | 2.827976845 | 4.548171117 | 0.685516226 | 6.55E-13 | 4.62E-12 | Up-regulated |
| ITPA | 12.03269951 | 19.21906002 | 0.675577433 | 4.45E-07 | 1.16E-06 | Up-regulated |
| CDIPT | 11.37845679 | 18.16053514 | 0.674501811 | 2.13E-12 | 1.35E-11 | Up-regulated |
| POLR2D | 3.995393916 | 6.373530231 | 0.673754938 | 1.20E-13 | 9.59E-13 | Up-regulated |
| DEGS1 | 16.69230629 | 26.36651079 | 0.659523366 | 8.37E-07 | 2.08E-06 | Up-regulated |
| AMDHD1 | 0.987611725 | 1.558769733 | 0.658391954 | 0.000146097 | 0.000264012 | Up-regulated |
| GPI | 23.6282004 | 37.26218069 | 0.657202354 | 6.45E-06 | 1.39E-05 | Up-regulated |
| EHHADH | 2.504802234 | 3.94670278 | 0.655951176 | 6.42E-07 | 1.63E-06 | Up-regulated |
| HAGHL | 1.090560767 | 1.718317495 | 0.655926468 | 1.09E-12 | 7.23E-12 | Up-regulated |
| LCAT | 1.67644694 | 2.632864902 | 0.651226674 | 1.30E-08 | 4.22E-08 | Up-regulated |
| PAFAH1B2 | 7.358224816 | 11.54857642 | 0.650285362 | 5.59E-10 | 2.35E-09 | Up-regulated |
| PFKP | 15.04065469 | 23.42551621 | 0.639213473 | 1.81E-05 | 3.69E-05 | Up-regulated |
| HEXA | 2.515425834 | 3.916119375 | 0.63862209 | 1.60E-10 | 7.36E-10 | Up-regulated |
| TPMT | 5.742226097 | 8.938103674 | 0.638358642 | 6.69E-10 | 2.77E-09 | Up-regulated |
| INPP5E | 2.727164408 | 4.244067526 | 0.638045934 | 6.64E-09 | 2.28E-08 | Up-regulated |
| DHDH | 1.225009344 | 1.901623657 | 0.634439003 | 5.42E-07 | 1.39E-06 | Up-regulated |
| NME2 | 14.6750624 | 22.74519849 | 0.632195387 | 1.44E-07 | 4.13E-07 | Up-regulated |
| TDO2 | 1.190929095 | 1.842573664 | 0.629634777 | 6.25E-15 | 6.36E-14 | Up-regulated |
| AGPS | 4.166335155 | 6.408103709 | 0.621118597 | 8.90E-10 | 3.58E-09 | Up-regulated |
| TAZ | 2.825331733 | 4.343729888 | 0.620514123 | 1.50E-09 | 5.89E-09 | Up-regulated |
| ENTPD6 | 6.009623852 | 9.226868782 | 0.618566446 | 3.47E-07 | 9.22E-07 | Up-regulated |
| MARS | 8.328784614 | 12.76485462 | 0.615999217 | 1.39E-11 | 7.54E-11 | Up-regulated |
| POLD3 | 2.661277628 | 4.075182795 | 0.614745752 | 3.28E-07 | 8.79E-07 | Up-regulated |
| AGPAT4 | 1.566557975 | 2.394800853 | 0.612307528 | 6.70E-08 | 1.97E-07 | Up-regulated |
| NIT2 | 3.00613399 | 4.584721136 | 0.60892467 | 5.78E-10 | 2.42E-09 | Up-regulated |
| PRPS2 | 9.326180295 | 14.21696039 | 0.608254822 | 0.000507594 | 0.000834657 | Up-regulated |
| ACP1 | 7.873679551 | 11.98992995 | 0.606713327 | 9.49E-12 | 5.28E-11 | Up-regulated |
| AK2 | 10.59189653 | 16.11522757 | 0.605463628 | 3.02E-09 | 1.12E-08 | Up-regulated |
| ACP2 | 6.518229781 | 9.821287912 | 0.591432013 | 7.60E-07 | 1.90E-06 | Up-regulated |
| PCK2 | 4.933289711 | 7.401728376 | 0.58531218 | 0.000262993 | 0.000451207 | Up-regulated |
| PIP4K2A | 3.123919942 | 4.645888645 | 0.572597094 | 2.60E-08 | 8.06E-08 | Up-regulated |
| INPP4A | 1.583888326 | 2.353690292 | 0.571453877 | 9.46E-13 | 6.42E-12 | Up-regulated |
| POLA1 | 2.478611497 | 3.680054214 | 0.570194862 | 2.68E-06 | 6.04E-06 | Up-regulated |
| ADSS | 9.55616775 | 14.16857541 | 0.568190624 | 4.37E-06 | 9.51E-06 | Up-regulated |
| ADK | 9.022099433 | 13.36788203 | 0.567235815 | 1.61E-06 | 3.78E-06 | Up-regulated |
| RDH11 | 7.232030941 | 10.68382223 | 0.562955121 | 1.42E-10 | 6.63E-10 | Up-regulated |
| PAPSS2 | 2.876679915 | 4.248912118 | 0.562688801 | 0.002500715 | 0.00374844 | Up-regulated |
| PISD | 4.032124847 | 5.90670809 | 0.550814006 | 9.54E-09 | 3.19E-08 | Up-regulated |
| DGKQ | 3.43557564 | 5.028636165 | 0.549615328 | 1.20E-06 | 2.92E-06 | Up-regulated |
| PIPOX | 0.981912955 | 1.428629065 | 0.540964336 | 1.55E-10 | 7.20E-10 | Up-regulated |
| CA12 | 16.67485271 | 24.21262672 | 0.538085581 | 0.028249038 | 0.035286517 | Up-regulated |
| LIPG | 1.241014818 | 1.79815557 | 0.534997501 | 0.00017517 | 0.000313369 | Up-regulated |
| NADSYN1 | 3.142229135 | 4.540838815 | 0.531170439 | 0.030007684 | 0.037417638 | Up-regulated |
| GMPPA | 4.291289027 | 6.180658858 | 0.526349565 | 1.75E-09 | 6.80E-09 | Up-regulated |
| PFKL | 11.53190243 | 16.60221847 | 0.525745499 | 1.17E-06 | 2.85E-06 | Up-regulated |
| POLR2J | 12.80943747 | 18.38215784 | 0.52109901 | 0.000741895 | 0.001203256 | Up-regulated |
| MTAP | 2.359781557 | 3.375130358 | 0.516289908 | 5.86E-05 | 0.000112074 | Up-regulated |
| PFKFB3 | 10.55330186 | 15.08436312 | 0.515359334 | 0.001412329 | 0.002190802 | Up-regulated |
| POLR1A | 3.349086218 | 4.769009665 | 0.50992219 | 2.84E-07 | 7.78E-07 | Up-regulated |
| GUSB | 7.390588172 | 10.51103125 | 0.508143132 | 9.38E-05 | 0.000174003 | Up-regulated |
| PFAS | 2.479993886 | 3.524563473 | 0.507108023 | 2.99E-07 | 8.11E-07 | Up-regulated |
| PGM3 | 3.133590864 | 4.451322019 | 0.506417045 | 3.96E-09 | 1.42E-08 | Up-regulated |
| ENTPD1 | 2.052876217 | 2.915503301 | 0.506098317 | 7.43E-09 | 2.52E-08 | Up-regulated |
| AGPAT3 | 7.948747647 | 5.613905081 | -0.501722906 | 0.000132475 | 0.000241234 | Down-regulated |
| CAT | 14.81468208 | 10.40660266 | -0.509528503 | 0.004721321 | 0.006763743 | Down-regulated |
| EPHX1 | 39.25752549 | 27.53771927 | -0.511560161 | 3.14E-08 | 9.54E-08 | Down-regulated |
| GBE1 | 7.469107479 | 5.2363406 | -0.512376917 | 1.07E-08 | 3.52E-08 | Down-regulated |
| CA8 | 1.501978289 | 1.046749641 | -0.520947535 | 1.92E-08 | 6.08E-08 | Down-regulated |
| ARG2 | 4.930102401 | 3.416967297 | -0.528901172 | 3.32E-05 | 6.59E-05 | Down-regulated |
| CMPK1 | 35.37607484 | 24.33208549 | -0.539914004 | 1.38E-08 | 4.45E-08 | Down-regulated |
| PIK3C2B | 4.487867786 | 3.073623132 | -0.546089893 | 1.54E-06 | 3.62E-06 | Down-regulated |
| DDO | 1.861093482 | 1.26842106 | -0.553116787 | 0.000782591 | 0.001260644 | Down-regulated |
| CHDH | 1.960899708 | 1.32381419 | -0.566815109 | 1.30E-06 | 3.14E-06 | Down-regulated |
| NAGK | 9.830848692 | 6.634327443 | -0.56736575 | 9.75E-06 | 2.04E-05 | Down-regulated |
| RETSAT | 16.00710668 | 10.77768107 | -0.57066576 | 3.53E-09 | 1.28E-08 | Down-regulated |
| ACSS2 | 9.697081669 | 6.480900296 | -0.581356395 | 1.10E-06 | 2.69E-06 | Down-regulated |
| CYP2E1 | 2.031067126 | 1.345859967 | -0.593709611 | 4.15E-08 | 1.25E-07 | Down-regulated |
| PCCA | 3.488990683 | 2.308720423 | -0.595716266 | 2.91E-14 | 2.62E-13 | Down-regulated |
| ME3 | 2.337933147 | 1.546677529 | -0.59606124 | 3.14E-09 | 1.15E-08 | Down-regulated |
| ACOX1 | 8.975917038 | 5.889638554 | -0.607880243 | 3.62E-09 | 1.31E-08 | Down-regulated |
| PLA2G3 | 5.376608725 | 3.521482558 | -0.610513548 | 0.008704101 | 0.011804419 | Down-regulated |
| ETNK2 | 6.714452889 | 4.393264112 | -0.611976617 | 0.018831561 | 0.023985816 | Down-regulated |
| ACAA1 | 6.454087667 | 4.214378169 | -0.614893397 | 6.48E-14 | 5.43E-13 | Down-regulated |
| ACO2 | 24.64656415 | 16.07202881 | -0.616834486 | 0.000739065 | 0.001201403 | Down-regulated |
| ADH1C | 5.338792594 | 3.477754181 | -0.618357541 | 2.15E-12 | 1.35E-11 | Down-regulated |
| ACYP2 | 3.023715447 | 1.961455623 | -0.624397682 | 3.63E-05 | 7.13E-05 | Down-regulated |
| HNMT | 2.956416886 | 1.915145418 | -0.626395778 | 2.25E-09 | 8.48E-09 | Down-regulated |
| ACADS | 8.732424314 | 5.612072305 | -0.637848637 | 0.003692366 | 0.005387223 | Down-regulated |
| SUOX | 4.749935895 | 3.038473021 | -0.644561561 | 1.56E-10 | 7.21E-10 | Down-regulated |
| PDHB | 10.52825445 | 6.717984943 | -0.648165796 | 1.24E-13 | 9.79E-13 | Down-regulated |
| CDO1 | 2.082478563 | 1.324854565 | -0.652467647 | 1.14E-09 | 4.52E-09 | Down-regulated |
| TYR | 1.5467369 | 0.979680404 | -0.658844726 | 0.007926055 | 0.010852599 | Down-regulated |
| HAL | 2.50865632 | 1.583167477 | -0.664100958 | 0.005683795 | 0.008029488 | Down-regulated |
| UGT1A8 | 2.068423125 | 1.287062831 | -0.684448856 | 1.52E-06 | 3.59E-06 | Down-regulated |
| ALDH3A2 | 21.75145735 | 13.43095962 | -0.695549678 | 7.86E-09 | 2.65E-08 | Down-regulated |
| ALAD | 9.923517673 | 6.09979193 | -0.702091584 | 7.29E-09 | 2.48E-08 | Down-regulated |
| ACOX3 | 7.70126558 | 4.732355453 | -0.702537109 | 7.90E-12 | 4.46E-11 | Down-regulated |
| ACADVL | 48.99355322 | 29.81584311 | -0.716512793 | 5.27E-12 | 3.05E-11 | Down-regulated |
| OAT | 31.13882724 | 18.92894535 | -0.718120578 | 5.26E-09 | 1.84E-08 | Down-regulated |
| SMPD2 | 8.695367603 | 5.28190619 | -0.719188341 | 1.01E-08 | 3.36E-08 | Down-regulated |
| HADHB | 30.02463622 | 18.23797214 | -0.71920144 | 3.22E-05 | 6.40E-05 | Down-regulated |
| GYS2 | 1.676297778 | 1.010292734 | -0.730505076 | 3.44E-17 | 6.27E-16 | Down-regulated |
| CA13 | 2.557685682 | 1.537985529 | -0.733797051 | 1.62E-13 | 1.23E-12 | Down-regulated |
| SMPD3 | 2.471295088 | 1.470588421 | -0.748873757 | 2.71E-06 | 6.08E-06 | Down-regulated |
| FMO1 | 3.556122579 | 2.109122901 | -0.75366189 | 4.45E-07 | 1.16E-06 | Down-regulated |
| GLUL | 72.81954232 | 42.8234754 | -0.765923789 | 3.05E-06 | 6.78E-06 | Down-regulated |
| SHMT1 | 7.357182829 | 4.291610251 | -0.777634382 | 4.10E-10 | 1.74E-09 | Down-regulated |
| PGD | 105.2178205 | 61.05643665 | -0.785163772 | 2.79E-06 | 6.25E-06 | Down-regulated |
| ALDH2 | 14.99267672 | 8.662211349 | -0.7914507 | 2.80E-12 | 1.72E-11 | Down-regulated |
| NT5C2 | 9.31798255 | 5.336892409 | -0.804017702 | 4.88E-09 | 1.72E-08 | Down-regulated |
| RDH12 | 8.279012087 | 4.73758833 | -0.805305783 | 0.008335221 | 0.011347375 | Down-regulated |
| UGT1A7 | 4.476170695 | 2.558381988 | -0.807033367 | 2.41E-06 | 5.49E-06 | Down-regulated |
| IDH2 | 69.85549617 | 39.80232919 | -0.811520772 | 1.22E-06 | 2.95E-06 | Down-regulated |
| UGP2 | 19.25038365 | 10.93276562 | -0.816228797 | 3.00E-07 | 8.13E-07 | Down-regulated |
| ALDH6A1 | 4.282590031 | 2.430583699 | -0.81718076 | 1.61E-10 | 7.36E-10 | Down-regulated |
| CP | 6.367253911 | 3.553779459 | -0.841317144 | 2.03E-07 | 5.70E-07 | Down-regulated |
| HADH | 18.6395783 | 10.2686278 | -0.860125814 | 2.77E-10 | 1.20E-09 | Down-regulated |
| MGST2 | 15.44507403 | 8.460779642 | -0.868284271 | 7.49E-14 | 6.13E-13 | Down-regulated |
| NME5 | 1.844204317 | 1.005017187 | -0.875778325 | 1.23E-09 | 4.83E-09 | Down-regulated |
| ASAH1 | 22.81348702 | 12.42042074 | -0.877172932 | 3.52E-13 | 2.53E-12 | Down-regulated |
| ARG1 | 3.846755909 | 2.08941087 | -0.880546071 | 0.004736777 | 0.006772259 | Down-regulated |
| SUCLG2 | 15.12412474 | 8.169654936 | -0.888504604 | 2.25E-16 | 3.20E-15 | Down-regulated |
| MGST1 | 8.938816094 | 4.805220068 | -0.895481261 | 8.19E-07 | 2.04E-06 | Down-regulated |
| NNT | 9.751400854 | 5.191399998 | -0.909485835 | 0.006606852 | 0.009169744 | Down-regulated |
| ACACB | 2.64260937 | 1.391791395 | -0.925020189 | 8.08E-08 | 2.35E-07 | Down-regulated |
| PGM1 | 23.04114858 | 12.06043365 | -0.933930854 | 0.01675751 | 0.021575673 | Down-regulated |
| ALOX15B | 11.10715028 | 5.720427083 | -0.957293951 | 0.016549276 | 0.021346168 | Down-regulated |
| PLCB4 | 2.608459317 | 1.335274107 | -0.966062001 | 0.000148596 | 0.000267849 | Down-regulated |
| INMT | 2.8356583 | 1.451482474 | -0.966156545 | 1.00E-06 | 2.47E-06 | Down-regulated |
| ACAT1 | 8.084459224 | 4.120766226 | -0.972238653 | 4.97E-09 | 1.74E-08 | Down-regulated |
| AOC3 | 3.927765911 | 1.989632624 | -0.981206881 | 0.036899726 | 0.045141933 | Down-regulated |
| GSTA1 | 29.40693272 | 14.89110123 | -0.981705864 | 1.36E-06 | 3.25E-06 | Down-regulated |
| CYP2J2 | 4.32893169 | 2.174071363 | -0.993611738 | 5.70E-18 | 1.45E-16 | Down-regulated |
| CES2 | 29.75584168 | 14.87288272 | -1.000488623 | 1.95E-06 | 4.56E-06 | Down-regulated |
| GOT1 | 33.54272058 | 16.52102827 | -1.021696225 | 2.51E-09 | 9.41E-09 | Down-regulated |
| ENPP3 | 2.115076669 | 1.024018991 | -1.046467489 | 7.40E-08 | 2.16E-07 | Down-regulated |
| ALDH3B1 | 4.29900603 | 2.073271769 | -1.052093893 | 1.70E-05 | 3.49E-05 | Down-regulated |
| ACADSB | 5.054004661 | 2.42496307 | -1.059464218 | 1.18E-13 | 9.55E-13 | Down-regulated |
| ALDH1A1 | 41.90379381 | 20.01285284 | -1.066154027 | 3.93E-12 | 2.32E-11 | Down-regulated |
| ACSL1 | 22.04481135 | 10.45351005 | -1.076451683 | 2.14E-09 | 8.14E-09 | Down-regulated |
| GATM | 5.309298458 | 2.502888523 | -1.084927208 | 3.94E-12 | 2.32E-11 | Down-regulated |
| AGL | 8.604761681 | 4.034325233 | -1.092807842 | 0.000262993 | 0.000451207 | Down-regulated |
| ALDH9A1 | 37.24567692 | 17.33632048 | -1.103275254 | 1.46E-17 | 3.36E-16 | Down-regulated |
| ACADM | 11.48270561 | 5.339513266 | -1.104682476 | 2.75E-09 | 1.02E-08 | Down-regulated |
| DCT | 2.182275508 | 1.011970871 | -1.108665487 | 1.35E-12 | 8.87E-12 | Down-regulated |
| PTGIS | 3.796156746 | 1.741739354 | -1.124010818 | 3.45E-05 | 6.82E-05 | Down-regulated |
| MAOB | 9.160535817 | 4.193501659 | -1.12727656 | 3.48E-11 | 1.80E-10 | Down-regulated |
| CHPT1 | 6.479311636 | 2.914507842 | -1.152588265 | 2.04E-10 | 9.07E-10 | Down-regulated |
| DEGS2 | 5.240332216 | 2.34458001 | -1.160328763 | 2.02E-10 | 9.06E-10 | Down-regulated |
| EPHX2 | 7.563168023 | 3.361848045 | -1.169736154 | 4.06E-17 | 7.05E-16 | Down-regulated |
| GNE | 8.76093777 | 3.723432954 | -1.234451924 | 2.09E-06 | 4.85E-06 | Down-regulated |
| AOX1 | 3.437701772 | 1.443790324 | -1.251583153 | 5.51E-15 | 5.69E-14 | Down-regulated |
| AMY1B | 2.38463981 | 0.996571129 | -1.258726686 | 0.005886392 | 0.008250218 | Down-regulated |
| GMDS | 10.89068409 | 4.476205492 | -1.282746405 | 9.39E-13 | 6.42E-12 | Down-regulated |
| ALDH1A3 | 11.25729793 | 4.588360893 | -1.294809807 | 7.23E-06 | 1.54E-05 | Down-regulated |
| GMPR | 6.791640373 | 2.739208386 | -1.310001044 | 0.005832194 | 0.008190379 | Down-regulated |
| ACHE | 4.749720349 | 1.899527348 | -1.322202091 | 0.000575195 | 0.000943638 | Down-regulated |
| CYP3A5 | 3.685299839 | 1.376553461 | -1.420721363 | 2.28E-08 | 7.12E-08 | Down-regulated |
| CYP2C18 | 9.557697005 | 3.496469103 | -1.450764276 | 3.34E-07 | 8.90E-07 | Down-regulated |
| GPT2 | 17.03872932 | 6.206746859 | -1.456908537 | 5.26E-19 | 1.78E-17 | Down-regulated |
| ACPP | 7.769603561 | 2.817494063 | -1.463428418 | 3.86E-10 | 1.65E-09 | Down-regulated |
| ADH7 | 39.64003621 | 14.31714504 | -1.469214444 | 4.16E-06 | 9.09E-06 | Down-regulated |
| SULT2B1 | 50.30141493 | 18.06914036 | -1.477071111 | 1.85E-07 | 5.23E-07 | Down-regulated |
| GAMT | 19.00609555 | 6.599177205 | -1.526104124 | 5.35E-05 | 0.000103033 | Down-regulated |
| LPL | 6.217156705 | 2.146047526 | -1.534572917 | 0.002580099 | 0.003851216 | Down-regulated |
| PTGDS | 16.91627608 | 5.800371247 | -1.544194864 | 2.10E-09 | 8.06E-09 | Down-regulated |
| ALDH3A1 | 139.0471331 | 47.24804134 | -1.55724757 | 3.20E-07 | 8.59E-07 | Down-regulated |
| DHRS9 | 10.60640927 | 3.484534028 | -1.605898676 | 0.000120057 | 0.00022088 | Down-regulated |
| GSTA2 | 3.339474807 | 1.073199738 | -1.637702623 | 1.01E-05 | 2.11E-05 | Down-regulated |
| DGAT2 | 8.269660272 | 2.542499933 | -1.701580327 | 2.84E-05 | 5.67E-05 | Down-regulated |
| GGT6 | 21.28731928 | 6.525950399 | -1.705734355 | 1.32E-15 | 1.51E-14 | Down-regulated |
| LDHD | 8.500934399 | 2.577298155 | -1.721761982 | 1.64E-15 | 1.82E-14 | Down-regulated |
| MGLL | 19.55200738 | 5.799637879 | -1.753282006 | 2.50E-19 | 1.05E-17 | Down-regulated |
| CYP2F1 | 3.43970697 | 1.014399618 | -1.76165956 | 1.18E-14 | 1.13E-13 | Down-regulated |
| ACER1 | 5.576072316 | 1.619526623 | -1.783677087 | 3.54E-07 | 9.38E-07 | Down-regulated |
| GPT | 5.496716408 | 1.439700056 | -1.932801773 | 8.08E-17 | 1.34E-15 | Down-regulated |
| HMGCS2 | 4.147856439 | 1.076948644 | -1.945416507 | 9.87E-21 | 7.81E-19 | Down-regulated |
| ALOX15 | 7.511172828 | 1.891936951 | -1.989174182 | 0.000129674 | 0.000236739 | Down-regulated |
| ALOX12 | 15.06141836 | 3.770001904 | -1.998220481 | 2.89E-06 | 6.46E-06 | Down-regulated |
| GPD1L | 14.61733168 | 3.378815718 | -2.113090406 | 3.52E-22 | 7.24E-20 | Down-regulated |
| GPX3 | 79.8026219 | 16.3497835 | -2.287164615 | 3.35E-17 | 6.27E-16 | Down-regulated |
| PLA2G2A | 31.83336545 | 6.278390555 | -2.342073007 | 2.38E-19 | 1.05E-17 | Down-regulated |
| ADH1B | 5.429257034 | 1.030037588 | -2.398057802 | 1.36E-21 | 1.80E-19 | Down-regulated |
| AMPD1 | 10.21817762 | 1.477917066 | -2.7895007 | 1.33E-08 | 4.30E-08 | Down-regulated |
| GPD1 | 9.462103952 | 1.148953681 | -3.041840373 | 4.23E-19 | 1.58E-17 | Down-regulated |
| FBP2 | 10.33585142 | 1.221050337 | -3.081462656 | 0.019492994 | 0.024739772 | Down-regulated |
| FMO2 | 22.80014378 | 2.624344549 | -3.119013874 | 1.52E-21 | 1.80E-19 | Down-regulated |
| CKMT2 | 18.84162075 | 2.080214676 | -3.179118745 | 2.40E-10 | 1.06E-09 | Down-regulated |
| CKM | 911.7462647 | 56.73915365 | -4.006215848 | 6.75E-08 | 1.98E-07 | Down-regulated |
| CA3 | 48.73935166 | 2.070884953 | -4.556767747 | 3.63E-11 | 1.86E-10 | Down-regulated |
| PYGM | 84.81602846 | 2.780843818 | -4.930742305 | 1.41E-11 | 7.61E-11 | Down-regulated |
| CA6 | 48.49317031 | 0.330555311 | -7.196746072 | 1.78E-05 | 3.63E-05 | Down-regulated |
